# Glymphatic perivascular clearance and extracellular vesicles: neuroimaging & blood‐based biomarkers in mild cognitive impairment

**DOI:** 10.1002/alz70856_100920

**Published:** 2025-12-24

**Authors:** Ramirez Joel, Lauren Abby Woods, Jeng‐liang Wu, Stephanie Berberian, Austyn D Roseborough, Min Su Kang, Erin Gibson, Daniela Andriuta, Sandra E. Black, Manuel Montero‐Odasso, Shawn N Whitehead

**Affiliations:** ^1^ Dr. Sandra Black Centre for Brain Resilience & Recovery, Sunnybrook Research Institute, Toronto, ON, Canada; ^2^ Schulich School of Medicine & Dentistry, Western University, London, ON, Canada; ^3^ Department of Neurology, Amiens University Medical Center, Amiens, France; ^4^ Laboratoire de Neurosciences Fonctionnelles et Pathologies (UR UPJV 4559), Jules Verne University of Picardy, Amiens, France; ^5^ Division of Neurology, Department of Medicine, Sunnybrook Health Sciences Centre, Toronto, ON, Canada; ^6^ Dr. Sandra Black Centre for Brain Resilience & Recovery, Toronto, ON, Canada; ^7^ Schulich School of Medicine & Dentistry, Division of Geriatric Medicine, Western University, London, ON, Canada; ^8^ Western University, London, ON, Canada

## Abstract

**Background:**

Poor glymphatic clearance is associated with various neurodegenerative disorders. Diffusion tensor imaging analysis along the perivascular space (DTI‐ALPS) has been suggested as a non‐invasive technique to assess glymphatic function, with lower DTI‐ALPS indices having been linked to reduced glymphatic function. We evaluated the relationships between the DTI‐ALPS index, plasma circulating extracellular vesicles (EV), whole brain atrophy and executive function in mild cognitive impairment (MCI) and cognitively normal (CN) individuals.

**Methods:**

Study participants (CN=35, MCI=33) were recruited from the Gait & Brain Cohort Study at Western University. DTI data was preprocessed using FSL Diffusion Toolbox commands, then FA and diffusivity maps were registered to a template. DTI‐ALPS indices were calculated by extracting diffusion data from designated regions of interest. EV blood‐based biomarkers were also collected: Total EV, TMEM119, pTau181, GFAP, GAL3, TMEM119/pTau181, TMEM119/GAL3, GFAP/GAL3. T‐tests were used to compare CN vs. MCI and regression models were used to evaluate associations between DTI‐ALPS, perivascular spaces (PVS), atrophy via the brain parenchymal fraction (BPF), and executive function (Trail‐making‐tests B‐A). Age, sex, education, body mass index, white matter hyperintensities, and systolic blood pressure were included as covariates.

**Results:**

The mean and left DTI‐ALPS indices were significantly lower in the MCI group compared to CN (left: *p* <0.02, mean *p* = 0.05). Regression models revealed that within the CN cohort, only age was negatively associated with BPF (β=0.704, *p* <0.001), however in the MCI cohort the mean DTI‐ALPS index was strongly associated with BPF (β=0.786, *p* <0.001). In both CN and MCI, GFAP/GAL3 was associated with PVS (CN: β=‐0.739, *p* = 0.013; MCI: β=1.089, *p* = 0.003). Furthermore, regression models revealed a negative association between Left DTI‐ALPS index and executive function in both the CN (β=‐0.395 *p* = 0.025) and MCI cohorts (β=‐0.447, *p* = 0.02).

**Conclusion:**

These preliminary results suggest that glymphatic dysfunction estimated by the DTI‐ALPS index is associated with brain atrophy, where the left hemisphere DTI‐ALPS is associated with executive dysfunction in both CN and MCI. Additionally, GFAP/GAL3 may be a potential useful astrocytic EV biomarker to indicate dysfunction of perivascular space clearance. Future work will examine mediation models with these imaging and EV biomarkers in neurodegenerative clinical populations.

